# Physicians’ perception and attitude toward electronic medical record

**DOI:** 10.1186/2193-1801-3-63

**Published:** 2014-02-03

**Authors:** Parvin Lakbala, Kavoos Dindarloo

**Affiliations:** Health information management Research Center, Hormozgan University of Medical Sciences, Bandar Abbas, Iran; Hormozgan Environmental and Occupational Health Engineering Research Center, Hormozgan University of Medical Sciences, Bandar Abbas, Iran

**Keywords:** Electronic Medical Record (EMR), Attitude, Perception, Specialist physicians

## Abstract

Implementation of an electronic medical record (EMR) system increases efficiency of health services, quality of care and patient satisfaction. Successful implementation of EMRs depends on many factors. The path to quality improvement and financial gain with EMRs lies in getting the greatest number of Physicians to use the system. The main objective of this research is to explore physicians, attitude and perceptions of importance EMRs function, anticipated utilization of EMR functions and also issue impact EMRs. The cross-sectional study was conducted on 133 specialist physicians of three teaching hospitals of Hormozgan Medical Sciences University. The most important finding in this study was the Entry/Display of Diagnoses and Medications (96.3%) and Prescription Alerts drug-drug, allergy and dose checking and formulary management (96.2%) were of greatest importance to respondents. Nuclear medicine, Time trended Clinical Data Display, decision support capabilities, and medical management reporting generated percentage suggesting less than weekly usage. Only a small number of respondents addressed physicians’ resistance in compare to another issues impact on EMRs. Understanding physician perceptions and attitude will allow for the development of targeted education to demonstrate the advantages and implementation of EMRs in further and improve physician perceptions of EMRs.

## Introduction

With the increase in the demand for high quality medical services, the need for an innovative Hospital Information System (HIS) has become essential (Yoo et al. 2008). HIS and electronic medical records (EMR) are considered prerequisites for the efficient delivery of high quality care and instrumental to the decrease in medical errors in healthcare delivery (Wang et al. 2003; Lium et al. 2008). It is widely acknowledged that the EMR has the potential to become the core electronic information and communication system in the healthcare sector (Ball 2003; Haux 2006; Chang and Chang 2008). The electronic medical record (EMR) is an enabling technology that allows physician practices to pursue more powerful quality improvement programs than is possible with paper-based records. However, achieving quality improvement through EMR use is neither low-cost nor easy (Miller and Sim 2004). It is widely believed that increased use of electronic medical records (EMRs) will improve the quality of health care and the efficiency with which it is delivered (Chaudhry et al. 2006; Sequist et al. 2007).

The path to quality improvement and financial gain with EMRs lies in getting the greatest number of Physicians to use the system (Miller and Sim 2004). Studies have been demonstrated that IT contributes to medical error prevention in the following categories; improved communication; more readily accessible knowledge; requirement for key pieces of information (such as the dose of a drug); assistance with calculations; checks performed in real time; assistance with monitoring; decision support; and rapid response to and tracking of adverse events (Bates and Gawande 2003). Of all the health information technology (IT) in current use, the electronic medical record (EMR) has the most wide-ranging capabilities and thus the greatest potential for improving quality. Research has demonstrated the quality benefits of electronic documentation and viewing, prescription and test ordering, care management reminders, and messaging, among other EMR functions (Balas 1996; Hunt et al. 1998). Despite this potential for quality improvement, however, few physician practices use EMRs. Nevertheless, interest in EMRs is substantial (Lynam and Karlan 2002; Harris Interactive 2003; American Academy of Family Physicians 2003).

With the assumption that EMR system can improve both the quality and effectiveness of the healthcare delivery, many healthcare provider organisations in developed countries have invested in the development and deployment of such systems (Lium et al. 2006). However, in many developing countries the EMR system is not widely disseminated or implemented. Published literature shows low adoption and high failure rate of successful EMR implementations (Ball 2003; Southon et al. 1999; Benson 2002; Littlejohns et al. 2003; Ash et al. 2004). Van der Meijden et al. (2003) identified user resistance as one of the primary factors for unsuccessful EMR implementation. Despite their potential advantages, implementation of EMR systems may be resisted if users are not satisfied with the system (van der Meijden et al. 2003). Although there is evidence of immediate benefit (Berg et al. 1998) and the improvements in time efficiency (Poissant et al. 2005), other factors, such as deficiencies in users’ computer skills (Dansky et al. 1999; van der Meijden et al. 2001; Ammenwerth et al. 2003) and the implementation process (Southon et al. 1999; Berg 1999; Aarts et al. 2004Berg 1999 also had a negative impact on successful EMR implementation (Berg 1999).

According to prior studies the slow rate of adoption suggests that resistance among physicians must be strong because physicians are the main frontline user-group of EMRs. Whether or not they support and use EMRs will have a great influence on other user-groups in a medical practice, such as nurses and administrative staff. As a result, physicians have a great impact on the overall adoption level of EMRs. As it requires physicians to actively support and use EMRs to benefit from them, it is essential to understand the possible barriers to their implementation from the physicians’ perspectives.

Incorporating a system with a main goal of making integrity between different organizations is not an easy task and many challenges and problems should be considered to make the system efficient for the organization. Although many good attempts have be done in some developed countries such as USA and some European countries there is still a lack of good framework for developing EMR and this issue is still a big challenge for many developing countries such as Iran. The present study broadly examined specialists physician affiliated in the HUMS perceptions related EMRs in the Bandar Abbas city of Iran. Understanding physician perceptions and attitude will allow for the development of targeted education to demonstrate the advantages and implementation of EMRs and improve further physician perceptions of EMRs.

## Methods

### Sample

To examine physician perceptions related to EMRs, a cross-sectional survey of 133 specialist physicians affiliated with the Hormozgan University of Medical Sciences (HUMS) in Bandar Abbas city of Iran was conducted. This study was carried out of 133 specialist physicians at three teaching hospitals of Hormozgan University of Medical Sciences (HUMS). That serve as tertiary referral centers and all of them are the major teaching hospitals affiliates of the HUMS. The study was approved by local ethical committee of HUMS and informed consent was obtained from all participants in this study. Questionnaires were sending to physician office and hospital wards in October 2012. The reminders were conveyed via follow up physician office visit. Of total 151 specialized physicians affiliated with the HUMS (88%) responses. There were 133 (88%) usable questionnaires. Participants included 133 of specialized physicians affiliated with the HUMS. Forty two (31.6%) of participants were female and 91(68.4%) were male. Participants had been employed at their practice sites from 2 to 24 years (Table 1).

**Table 1:** **Respondents, profile**

Variable		No(%)
**Age**		
	<35	32(24.1)
	35-50	89(66.9)
	>50	12(9.0)
**Gender**		
	Male	91(68.4)
	Female	42(31.6)
**Specialty**		
	Medical specialties (Dermatologist, Cardiologist, Internal Medicine, Neurologist)	29(22.60)
	Psychiatry	5(3.8)
	Pediatrics	19(14.3)
	Surgery (Neuro Surgery, *GYN/OB, Surgery,** ENT, Ophthalmologist, Urologist, Orthopaedics)	51(38.30)
	Others (Radiologists, Anesthesiologists, Pathologists,…)	29(21.8)
		133(100.0)

*OB/GYN: obstetrics & gynaecology.

** E.N.T: ears, nose and throat.

The physicians specialized in different areas including Medical specialties (Dermatologist, Cardiologist, internal medicine, neurology,) Psychiatry, Pediatrics, Surgery (Neuro Surgery, GYN/OB, Surgery, ENT, Ophthalmologist, Urologist, Orthopedics) and others (Radiologists, Anesthesiologists, Pathologists,…).

### Questionnaire

The authors designed a multi-section questionnaire based on previous EMR research and Meinert study (29). Individual sections of the questionnaire addressed; Respondents^,^ access to computer and clinical data required respondents’ attitude and perceptions regarding importance of EMR functions and anticipated utilization of EMR functions. Five physicians with expertise in medical informatics screened the questionnaire for content validity. Fifteen specialist physicians reviewed the instrument for structure, clarity, and relevance to test face validity. Ten physicians generated a test–retest reliability rate of >80% for each item over a 2-week interval.

The questionnaire consisted of 2 parts. The first section included questions about physician demographics (age, gender, specialty and works age). The second section included questions about respondents’ perception and attitudes related to EMRs using a Likert scale ranging from “agree” to “disagree”. The Likert scales were collapsed to a dichotomous variable, “agree”,”slightly agree”, “disagree” and “no comment” for this analysis. And also a six point Likert scale from Very Important to Very Unimportant was used to determine respondents’ Anticipated Utilization of EMR Functions. Questionnaires were sending in October 2012, with a follow up visiting to non respondents 2 weeks later. The responses were entered into a spreadsheet and the data entry was verified for accuracy via manual verification. The collected data were analyzed using SPSS v. 12 (SPSS, Chicago, IL, USA) and *χ*2 test, with *P*<0.05 considered statistically significant. The percentages and their 95% confidence intervals are presented.

## Results and discussion

The results of the survey were tabulated and percentile analysis was carried out. (Table 2 summarizes respondents’ access to computer, training and requirements related to inpatient vs. out-patient data. A significantly greater proportion (94% vs. 86%) of physicians reported requiring access to inpatient versus outpatient data. About one-half (50.4%) of the respondents expressed that they used computer daily and only 23(17.3%) of physicians haven’t used of computer. Physicians skills in use of computer was 53(38.9%) good, 64(48.1%) moderate and only 16(12.1%) of them have less skill in use computer.

**Table 2:** **Respondents** ^**,**^ **access to computer and clinical data required**

Variable	Yes	No
Own a computer	121(91.0)	12(9.0)
Place of access to computer		
Office	58(43.6)	75(56.4)
Hospital	81(60.9)	52(39.1)
Formal computer training	72(54.1)	61(45.9)
Clinical data required		
Inpatient	125(94.0)	8(62.4)
Outpatient	115(86.5)	18(13.5)

(Table 3 summarizes the respondents’ perceptions regarding the importance of specific EMR functions. Respondents were presented with a list of nineteen functions associated with what has been described as a Second Generation EMR (Ammenwerth et al. 2003). Respondents considered all of the EMR functions presented to be at least slightly important. As noted in (Table 3, the Entry/Display of Diagnoses and Medications (96.3%) and Prescription Alerts drug-drug, allergy and dose checking and formulary management (96.2%) were of greatest importance to respondents. Despite this interest in accessing such data, respondents rated a display of Height, Weight and Allergies (84.2%) and also a Nuclear Medicine (76.0%) results as one of the least important functions. Display of prescription alerts was considered slightly more important (96.2%) than the ability to prescribe online (94.5%). Structured documentation and physician order entry (POE) received (92.4%) and (94.7%), respectively. Despite the promise of EMRs to offer health reminders and decision support at the point of care these functions along with medical management reporting were rated the least important by respondents. This finding echoed by meinert study (Meinert 2005).

**Table 3:** **Respondents’ perceptions regarding the importance of specific EMR functions**

Function	Very important	Important	Slightly important	Ordinary	No important	Very unimportant
Display of lab results	88(66.2)	31(23.3)	7(5.3)	7(5.3)	-	-
Display of radiology reports	64(48.1)	41(30.8)	17(12.8)	6(4.5)	3(2.3)	2(1.5)
Display of clinical notes and reports	68(51.1)	41(30.8)	16(12.0)	5(3.8)	3)2.3)	-
Display of height, weight and allergies	44(33.1)	51(38.3)	17(12.8)	17(12.8)	4(3.0)	
Nuclear medicine	30(22.6)	48(36.1)	23(17.3)	15(11.3)	6(4.5)	11(8.3)
Display of radiology images	56(42.1)	42(31.6)	22(16.5)	9(6.8)	3(2.3)	1(0.8)
Entry/Display of diagnoses and medications	73(54.9)	44(33.1)	11(8.3)	-	3(2.3)	2(1.5)
Display of other ancillary clinical data	35(26.3)	46(34.6)	38(28.6)	11(8.3)	2(1.5)	1(0.8)
Prescription alerts drug-drug, allergy and dose checking and formulary management	61(45.9)	5.3(39.8)	14(10.5)	-	5(3.8)	-
Display of demographics	28(21.5)	56(42.1)	30(22.6)	15(11.3)	2(1.5)	2(1.5)
Time trended clinical data display	36(27.1)	60(45.1)	30(22.6)	-	7(5.3)	-
Structured documentation (Templates)	50(37.6)	53(39.8)	20(15.0)	9(6.8)	1(0.8)	-
Physician order entry (tests and medication orders)	67(50.4)	37(27.8)	22(16.5)	5(3.8)	2(1.5)	-
Prescription writing	59(44.4)	53(39.8)	15(11.3)	4(3.0)	2(1.5)	-
Workflow inbox for office and/or hospital results	40(30.1)	66(49.6)	16(12.0)	8(16.0)	2(1.5)	-
Decision support (guidelines, expert logic, reminders/alerts)	37(27.8)	47(35.3)	33(24.8)	14(10.5)	1(0.8)	1(0.8)
Preventative health reminders	42(31.6)	59(44.4)	17(12.8)	13(9.6)	1(0.8)	1(0.8)
Medical management reporting – notification by diagnoses	44(33.1)	55(41.4)	18(13.5)	13(9.8)	2(1.5)	1(0.8)
Medical management reporting – disease management reporting	38(29.3)	48(36.1)	33(24.8)	9(6.8)	3(2.3)	1(0.8)

(Table 4 summarizes respondents’ Anticipated Utilization of EMR Functions. Percentage responses for these items suggest more than 70% of respondents in this study expressed 6 of the 19 EMR functions would be used at least weekly. Not surprisingly, anticipated utilization of functions is highly correlated with perceived importance. Only 71(52.4%) of respondents anticipated at least utilization of function related to the Nuclear medicine less than weekly usage. Nuclear medicine, Time trended Clinical Data Display, decision support capabilities, and medical management reporting generated percentage suggesting less than weekly usage. For majority functions at least one-half of the respondents anticipated daily use for more and some, of their patients. Consistent with the importance ratings respondents’ anticipated greater utilization of functions related to the Prescription Writing 101(75.7%).

**Table 4:** **Anticipated Utilization of EMR Functions**

Function	More patient/daily	Some patient/daily	Weekly	Monthly	Every 3 Months	Never
Display of lab results	57(42.9)	29(21.8)	12(9.0)	15(11.3)	15(11.3)	5(3.8)
Display of radiology reports	45(33.8)	32(24.1)	15(11.3)	16(12.0)	17(12.8)	8(6.0)
Display of clinical notes and reports	51(38.3)	36(27.1)	10(7,5)	16(12.0)	11(8.3)	(6.8)
Display of height, weight and allergies	24(18.0)	46(34.6)	19(14.3)	23(17.3)	10(7.3)	11(8.3)
Nuclear medicine	19(14.3)	21(15.8)	31(23.3)	20(15.0)	21(15.8)	21(15.8)
Display of radiology images	40(30.1)	31(23.3)	19(14.3)	19(14.3)	12(9.0)	12(9.0)
Entry/Display of diagnoses and medications	47(35.3)	38(28.6)	8(6.0)	26(19.5)	8(6.0)	6(4.5)
Display of other ancillary clinical data	28(21.1)	45(33.8)	20(15.0)	15(11.3)	10(7.5)	15(11.3)
Prescription alerts drug-drug, allergy and dose checking and formulary management	26(19.5)	35(26.3)	24(18.0)	28(21.1)	13(9.8)	7(5.3)
Display of demographics	34(25.6)	34(25.6)	21(15.8)	23(17.3)	13(9.8)	8(6.0)
Time trended clinical data display	31(23.3)	22(16.5)	29(21.8)	11(8.3)	25(18.8)	15(11.3)
Structured documentation (Templates)	55(41.4)	28(21.1)	15(11.3)	20(15.0)	10(7.5)	5(3.8)
Physician order entry (tests and medication orders)	57(42.9)	31(23.3)	11(8.3)	17(12.8)	11(8.3)	6(4.5)
Prescription writing	52(39.1)	35(26.3)	14(10.5)	11(8.3)	15(11.3)	6(4.5)
Workflow inbox for office and/or hospital results	33(24.8)	32(24.1)	22(16.5)	14(10.5)	26(19.5)	6(4.5)
Decision support (guidelines, expert logic, reminders/alerts)	38(28.6)	27(20.3)	18(13.5)	20(15.0)	22(16.5)	8(6.0)
Preventative health reminders	41(30.8)	28(21.1)	18(13.5)	19(14.3)	19(14.3)	8(6.00)
Medical management reporting – notification by diagnoses	25(18.8)	38(28.6)	18(13.5)	22(16.5)	21(15.6)	9(6.8)
Medical management reporting – disease management reporting	31(23.3)	35(26.3)	12(9.0)	21(15.8)	28(21.1)	6(4.5)

(Table 5 summarizes respondents’ general attitudes and perceptions regarding EMRs, including familiarity with functions and benefits, impacts, usage/training and overall value and need for adoption. Both the frequent rating for each item and the percentages “agreeing” and “disagreeing” are presented. These percentages were calculated by collapsing a Likert scale from Agree to Disagree. The respondents’ general attitudes and beliefs (See (Table 5) suggest overall support for EMRs. A majority 103(77.0%) of the respondents expressed that physicians were familiar with EMR functions and benefits. Over three-quarters of the respondents agreed that EMRs would improve both quality of care 119(89.0%) and quality of practice (i.e., work life) 117(88.0%). To a lesser extent, respondents 104(85.7%) believe that practice productivity would increase with an EMR. This later perception may reflect in part physician experiences with early clinical systems that often offered little in the way of time savings (Treister 1998). Given physician concerns regarding productivity it is critical that they be motivated (e.g., incentives) to participate in training. This finding supported by other studies (Miller and Sim 2004; Silow Carroll et al. 2012; Hillestad et al. 2005). EMR technology offers a number of potential benefits, including improved quality of patient care, more efficient healthcare workflows, and reduced costs (Thompson et al. 2007). Improvement in the quality of patient care can be credited to several attributes of an EMR system including superior documentation, flexible data organization, integrated systems, and assisted clinical decision making (Shekellee et al. 2006). Recent study by Xiao and et al. showed that the use of core functionalities required by “meaningful use” criteria and the use of certified EMRs have a positive impact on the quality and efficiency of health care(Xiao et al. 2012). Gold and et al. conducted a improvement study using fishbone analysis and an Electronic Medical Records intervention to improve care for children with asthma and found that a systematic approach to quality improvement using plan-do-check-act (PDCA) and fishbone analysis in conjunction with embedded EMR tools can improve asthma care in a pediatric primary care setting (Gold et al. 2013). Nearly one-third 50 (38.0%) of the respondents expressed doubt that physicians would devote the time required for EMR training. The findings supported by prior study (Meinert 2005). Nearly one- half 65(48.8%) of respondents expected the EMRs induced hard work and a majority 117(88.0%) of them felt EMRs need spend time for training. The majority of respondents 112 (84.2%) felt that benefits of an EMR outweigh the costs and that an EMR should be implemented. The finding that more than three-quarters of the physicians felt usage would have to be mandated is consistent with Hier (2002) who recommended mandating EMR utilization (Hier 2002). Respondents were expressed general concerns regarding EMRs in a wide range of issues including: impact on cost, security, patient acceptance, privacy, complexity, response time for patient, training needs and physician resistance.

**Table 5:** **Perception and Attitudes Regarding EMRs**

Attitude/beliefs		Agree	Slightly agree	Disagree	No comment
Physicians are familiar with		24(18.0)	79(59.0)	18(14)	12(9.0)
EMR functions and benefits					
EMRs improve quality of care and reduce errors		63(47.0)	56(42.0)	4(3.0)	10(8.0)
EMRs improve quality of practice (i.e., work life)		61(45.9)	56(42.1)	5(3.8)	11(8.3)
EMRs increase practice productivity (i.e., patients per day)		69(51.9)	45(33.8)	7(5.3)	12(9.0)
Physicians will devote the time required for EMR Training		33(24.9)	50(38.0)	40(30.0)	10(8.0)
EMRs induced hard work		18(13.5)	47(35.3)	54(40.6)	14(10.5)
EMRs need spent time for training		56(42.1)	61(45.9)	5(3.8)	11(8.3)
EMR benefits outweigh the costs		60(45.1)	52(39.1)	10(7.5)	11(8.3)
EMRs issue					
Impact on cost		40(30.1)	65(48.9)	19(14.3)	9(6.8)
Impact on security		55(41.4)	54(40.6)	15(11.3)	9(6.8)
Patient acceptance		55(41.4)	55(41.4)	12(9.0)	11(8.3)
Privacy		68(51.1)	39(29.3)	18(13.5)	8(6.9)
Complexity		39(29.3)	66(49.6)	12(9.0)	16(12.0)
Response time for patient		55(41.4)	54(40.6)	14(10.5)	10(7.5)
Training needs		65(48.9)	49(36.8)	4(3.0)	15(11.3)
Physicians resistance		28(21.1)	55(41.4)	33(24.8)	17(12.8)
An EMR system should be implemented		63(47.4)	49(36.8)	7(5.3)	14(10.5)

105(79.0%) of respondents expected the impact on costs. Funding and costs for implementation EMRs mentioned in prior studies (Miller and Sim 2004; Bates and Gawande 2003; Ash et al. 2004; Hillestad et al. 2005; Boudreau et al. 2005;Ford et al. 2005; Anderson 2004; Valdes et al. 2004). 109(82.0%) of respondents expressed use EMRs will impacted on security. 107(80.4%) of respondents expected the impact on privacy, this issue mentioned in many prior studies that shown their concerns about privacy and security of patient data as an issue to EMRs usage (Bates and Gawande 2003; Hillestad et al. 2005; Valdes et al. 2004; Soper 2002; Waegemann 2002; Jha et al. 2009; Menachemi et al. 2007; Loomis et al. 2002; Vishwanath and Scamurra 2007; Earnest et al. 2004; McLane 2005). EMR software should have the ability to limit access to various portions of the record to particular users. 105(78.9%) of respondents mentioned the complexity of EMRs as an issue for implementation system. This finding supported by other studies (Miller and Sim 2004; Burns 2001). 109(82.0%) felt response time for patient was as issue and 114(85.7%) training needs as an issue regarding EMRs implementation. A notable finding within the attitude assessment was the overwhelming percentage that agreed about EMRs issues that needs to be tackled and effective management should be a team effort. Physicians resistance mentioned by 83(62.5%) of respondents while patient acceptance mentioned by majority of respondents 110(82.8%). More than 80% of physicians both show their resistance to EMR and their positive attitude to implement EMRs. This is an interesting result that physicians resitance maybe due to EMRs issue, usefulness and ease-of-use of the technology than EMRs function and benefits. This finding echoed to Meinert study (Meinert 2005). Since the majority of physicians currently believe EMR benefits outweigh costs, it comes as no surprise that 84.2% percent feel an EMR system should be implemented.

## Conclusions

Health Care system has had an ongoing focus on improving access to and quality of care, and more recently on cost reduction. The primary mean to achieve these goals has been to change health care policy, as exemplified by the adoption of health information technology in particular the adoption of patient centred information, characterized by the ability to manage comprehensive patience information such as: medical records; appointments scheduling; theatre management and ward reporting. Despite the positive effects from using EMRs in medical practices, the adoption rate of such systems is still low and they meet resistance from physicians. Our data demonstrate the many different beliefs that physicians have about implementation EMRs. The EMR is an enabling technology for physician practices to pursue quality improvement in potentially powerful ways. There is no simple solution to accelerating EMR adoption and use for quality improvement. Given the multifaceted nature of the barriers, a range of policy interventions is needed to spur successful EMR-driven quality improvement. The success of health information technology (IT) depends a great deal on individual-level responses of clinician end users; those responses include acceptance/rejection of the IT and how (even whether) clinicians use the IT.

Given the obvious need for national standards and widespread integration, a government role in mandating and creating incentives for the healthcare industry to move in the direction of nationwide EMR would be required. Also worthy of further analysis is a global EMR system compatible with other countries and compliant with global standards. A range of policy options could be used to speed the development of EMR. Healthcare administrators need to begin preparing their staff for the inevitable technology upgrades that will take place in their practices. In an industry where there is less exposure to modern communication technology than most others, educational campaigns should be initiated. While there are many barriers to widespread adoption of EMRs those associated with physicians are not insurmountable. On the contrary, by systematically examining physician perceptions related to EMRs both vendors and healthcare organizations can accelerate physician acceptance and ultimately the rate of adoption and utilization. Understanding physician perceptions will allow for the development of targeted education to demonstrate the advantages of EMRs and further improvement physician perceptions of EMRs. Implementing an EMR system requires good planning, strong physician leadership and supportive staff.

Electronic medical record system requires the user and attributes, support from others, and numerous organizational and environment facilitators. In addition, difficulty of using EMRs and the non-use of specific functions result from the presence of barrier. Many theoretical models, such as Technology Acceptance Model, posit that users’ acceptance toward a specific technology depends on the perceived usefulness and ease-of-use of the technology. For the EMR systems to have a positive impact on patient safety, physicians must be able to use these systems effectively after they are made available. Many studies showed that implementers can insulate the project from such concerns by establishing strong leadership, using project management techniques, establishing standards and training their staff to ensure such risks do not compromise implementation success.
